# Multi-Agent Variational Approach for Robotics: A Bio-Inspired Perspective

**DOI:** 10.3390/biomimetics8030294

**Published:** 2023-07-07

**Authors:** Imran Mir, Faiza Gul, Suleman Mir, Laith Abualigah, Raed Abu Zitar, Abdelazim G. Hussien, Emad Mahrous Awwad, Mohamed Sharaf

**Affiliations:** 1 School of Avionics and Electrical Engineering, College of Aeronautical Engineering, NUST, Risalpur 23200, Pakistan; 2 Department of Electrical Engineering, Air University, Aerospace and Aviation Campus Kamra, Kamra 43600, Pakistan; faiza.gul@aack.au.edu.pk; 3 Department of Electrical Engineering, National University of Computer and Emerging Sciences, Peshawar 21524, Pakistan; suleman.mir@nu.edu.pk; 4 Computer Science Department, Prince Hussein Bin Abdullah Faculty for Information Technology, Al Al-Bayt University, Mafraq 25113, Jordan; 5 Hourani Center for Applied Scientific Research, Al-Ahliyya Amman University, Amman 19328, Jordan; 6 MEU Research Unit, Middle East University, Amman 11831, Jordan; 7 Applied Science Research Center, Applied Science Private University, Amman 11931, Jordan; 8 Sorbonne Center of Artificial Intelligence, Sorbonne University-Abu Dhabi, Abu Dhabi 38044, United Arab Emirates; raed.zitar@sorbonne.ae; 9 Department of Computer and Information Science, Linköping University, 58183 Linköping, Sweden; abdelazim.hussien@liu.se; 10 Electrical Engineering Department, College of Engineering, King Saud University, Riyadh 11421, Saudi Arabia; 442106835@student.ksu.edu.sa; 11 Industrial Engineering Department, College of Engineering, King Saud University, Riyadh 11421, Saudi Arabia; mfsharaf@ksu.edu.sa

**Keywords:** multi-agent, numerical optimization, space exploration, meta-heuristic, bio-inspired, augmented framework, Aquila Optimizer

## Abstract

This study proposes an adaptable, bio-inspired optimization algorithm for Multi-Agent Space Exploration. The recommended approach combines a parameterized Aquila Optimizer, a bio-inspired technology, with deterministic Multi-Agent Exploration. Stochastic factors are integrated into the Aquila Optimizer to enhance the algorithm’s efficiency. The architecture, called the Multi-Agent Exploration–Parameterized Aquila Optimizer (MAE-PAO), starts by using deterministic MAE to assess the cost and utility values of nearby cells encircling the agents. A parameterized Aquila Optimizer is then used to further increase the exploration pace. The effectiveness of the proposed MAE-PAO methodology is verified through extended simulations in various environmental conditions. The algorithm viability is further evaluated by comparing the results with those of the contemporary CME-Aquila Optimizer (CME-AO) and the Whale Optimizer. The comparison adequately considers various performance parameters, such as the percentage of the map explored, the number of unsuccessful runs, and the time needed to explore the map. The comparisons are performed on numerous maps simulating different scenarios. A detailed statistical analysis is performed to check the efficacy of the algorithm. We conclude that the proposed algorithm’s average rate of exploration does not deviate much compared to contemporary algorithms. The same idea is checked for exploration time. Thus, we conclude that the results obtained for the proposed MAE-PAO algorithm provide significant advantages in terms of enhanced map exploration with lower execution times and nearly no failed runs.

## 1. Introduction

Multi-Agent Robot Exploration is a field of study that deals with finding optimal solutions for a group of robots exploring an unknown environment. The main challenge in this field is to coordinate the actions of multiple robots so that they can work together effectively and efficiently [1,2,3,4,5,6,7,8,9,10,11,12]. In order to solve this problem, researchers often use optimization algorithms, which are mathematical methods that find the best solution to a given problem based on specific objectives and constraints [13,14,15,16,17,18,19,20,21,22,23,24,25,26,27,28]. Using a group of autonomous mobile robots can have a number of benefits, including increased effectiveness, dependability, and robustness when conducting tasks such as exploration, surveillance, and inspection to acquire information [29]. These benefits are attained by utilizing some kind of team coordination, which is frequently built assuming the ability to interact without boundaries. However, dealing with communication-challenged circumstances is a common requirement for operations in the real world. Robots in these environments can only communicate with teammates nearby (locally), depending on their transmission capabilities and the environment itself (e.g., the presence of obstacles or disturbances). It may not be easy to achieve a good level of coordination as a result.

Creating maps is one of the most fundamental yet difficult tasks for a group of autonomous mobile robots. Map creation is frequently a part of the problem-formulation and solution processes in applications in search and rescue, surveillance, and similar disciplines. As a result, many of the conclusions we reach in the following can be readily applied to other issues [30,31]. The scenario of multi-robot exploration with restricted communication has received far less attention than the one with unconstrained communication (see, for example, [32]). However, limiting communication has several drawbacks. The shared understanding of the surroundings throughout the exploratory journey is the first important problem. Such knowledge can be presumed to be available to each robot simultaneously with limitless communication. In fact, data-sharing protocols may be used by map-merging algorithms [33] (whether distributed or centralized) to exchange the updated map.

The frontier notion is often used in autonomous exploration methods to frame the issue. The idea of a frontier between open space and uncharted space serves as the foundation for the single-robot exploration approach, which was first put forth by [34]. A team of robots simultaneously exploring various areas of an unknown environment is the foundation of another coordinated multi-robot exploration approach [35]. The deterministic, well-liked method called Coordinated Multi-Exploration (CME) attempts to deterministically build a definable map in an unknowable space. The goal of an exploration procedure is to cover the complete environment in the shortest amount of time. Therefore, the robots must use a centralized method to continuously track which areas of the environment have already been explored. Since CME is deterministic and always repeats the same search pattern, there is no way to avoid local optima. The only way to solve this problem is to change the environment’s setting, which is not always possible. In addition, there is no certainty that a waypoint will be reached, which has a significant impact on the robots individually and causes them to forget their assigned tasks, which leads to a breakdown in coordination. For exploration, robots require the development of a global map in order to plan their paths and coordinate their operations [36]. The robots may always communicate with one another about where they are on the map, what they have looked at, and where they are currently on the map. The occupancy grid [37] is used by robots to communicate with one another.

The whole issue of exploration can be abstractly defined as follows: Suppose there is a setting space *S* and that time is discretized into steps. There are *n* robots in the collection *nRbt =* {r1, r2, r3, … rn }, each of which can move in *S*. Call $p_{j}^{t}\subseteq S$ the area of *S* that robot *j* is aware of at time *t*. Let us assume that every robot can always communicate with every other robot. The map of the area of the environment that robots are capable of perceiving at time *t* is provided by: $M^{t}=\cup_{j=1},\ldots \cup_{t=0,\ldots .t}*p_{i}^{t}$

When there occurs a period *t* such that $M^{t}$ = *S* (or such that $M^{t}$ is equal to the free space of *S*), the exploration is finished. The challenge of multi-robot exploration is to decide, while optimizing a performance metric, which frontiers (borders of the known area of the environment $M^{t}$) the robots should travel towards at each time step *t*. The time *t* to finish exploration (to be minimized), the area mapped in a given time (to be maximized), the distance traveled by robots to finish exploration (to be minimized), or combinations of those metrics are typical performance measures. The exploration approach, which incorporates the *“intelligence”* of the system, determines which frontier each robot should explore in order to optimize the performance measure.

### 1.1. Motivating Problem for the Paper

According to the publications cited in the literature study, hybridization is the newest trend in algorithms [38,39,40,41]. Different algorithms are combined to create hybrids that outperform their individual components while requiring fewer resources. Researchers are developing new hybrid algorithms with the express purpose of addressing the shortcomings of existing ones by enhancing the optimization variables and conducting experiments with varying degrees of complexity. Therefore, this method is critical to developing novel hybrid solutions that accommodate limited real estate and include bio-inspired methods in the MAE paradigm.

### 1.2. Research Contributions

Our study makes significant contributions to the field of space exploration in obstacle-cluttered environments by proposing a novel hybrid technique that combines deterministic and swarm-based methods. The key contributions of our approach include:

- **Hybrid Methodology:** We present a hybrid methodology that combines deterministic and swarm-based approaches, harnessing the benefits of each.
- **Adaptability:** Because of the incorporation of swarm-based methodologies, which demonstrate emergent behavior and decentralized decision-making, our technology is deemed versatile. This versatility allows the robots to adjust to changes in the environment and deal with unexpected impediments or interruptions.
- **Robustness:** The combination of deterministic and swarm-based approaches improves our approach’s overall robustness, allowing it to effectively navigate through complicated obstacle configurations.
- **Exploration Efficiency:** By integrating established exploration patterns from deterministic methods with real-time changes and optimization possibilities afforded by swarm-based approaches, we optimize exploration efficiency.

## 2. Related Studies

In the related-work section, the authors discuss several existing methods for multi-agent exploration and optimization and highlight their limitations [42].

The basic objective of an autonomous robotic exploration algorithm is to guide robots into uncharted terrain, expanding the known and explored portion of a map that is being constructed as the robot moves. The frontier notion is often used in autonomous exploration methods to frame the issue. In other words, it can also be explained that the goal of Multi-Agent Robot Exploration Optimization is to find an optimal exploration strategy for a team of robots that allows them to cover a large area of the environment, gather information, and avoid obstacles while using minimal resources such as time and energy [43,44,45,46]. One approach for multi-agent exploration is based on swarm intelligence, where a group of agents interacts with each other and their environment to achieve a common goal. Swarm intelligence algorithms, such as ant colony optimization and particle swarm optimization, have been applied to multi-agent exploration problems, but they can be limited by their sensitivity to the initial conditions and their inability to handle complex environments. Another approach is based on genetic algorithms, which are inspired by biological evolution and use a population of solutions to search for an optimal solution. Genetic algorithms have been applied to multi-agent exploration problems, but they can be computationally expensive and require a large number of evaluations to converge to an optimal solution.

This paper attempts to address the shortcomings of the ANFIS by using a modified Aquila Optimizer (AO) and the Opposition-Based Learning (OBL) technique to optimize ANFIS parameters. The major goal of the developed model, AOOBL-ANFIS, is to improve the Aquila Optimizer (AO) search process while utilizing the AOOBL to improve the ANFIS’s performance. Utilizing a variety of performance metrics, including root mean square error (RMSE), mean absolute error (MAE), coefficient of determination (R2), standard deviation (Std), and computational time, the proposed model is assessed using real-world oil production datasets gathered from various oilfields [47].

In [48], the authors presented the integration of reinforcement learning with a multi-agent system. To be more precise, the authors suggest knowledge normalization to optimize the reciprocal information between agents’ identities and their trajectories to promote thorough investigation and a range of unique behavioral patterns. To encourage learning sharing across agents while maintaining sufficient variety, the author added agent-specific modules in the shared neural network design. These modules are regularized using the L1-norm. Experimental findings demonstrate that the approach delivers state-of-the-art performance on highly challenging StarCraft II paternalistic management tasks and researching online football.

The approach proposed in [49] combines a parallel computational Aquila Optimizer, a bio-inspired technology, with deterministic multi-agent exploration. The Aquila Optimizer is inspired by the behavior of eagles and uses a stochastic search strategy to explore the search space efficiently. Stochastic factors are integrated into the Aquila Optimizer to enhance the algorithm efficiency. The proposed approach is compared with several existing approaches, including the whale algorithm, using a simulation of a multi-agent exploration problem. The results demonstrate that the proposed approach outperforms the existing approaches in terms of convergence speed and solution quality. Overall, the proposed approach offers a promising solution for multi-agent exploration problems, particularly in complex and uncertain environments, by leveraging bio-inspired optimization algorithms and integrating stochastic factors into the search strategy.

Albina et al. [50] suggest stochastic optimization to simulate the coordinated predatory behavior of grey wolves, and they apply it to multi-robot exploration. Here, deterministic and metaheuristic methods are coupled to compute the robots’ movement. A novel approach called ‘hybrid stochastic exploration’ makes use of the Coordinated Multi-Robot Exploration and Grey Wolf Optimizer algorithms. The obtained result shows the proposed algorithm performs better compared to other algorithms. However, no modification was found in the bio-inspired algorithm to check the performance of the Grey Wolf Optimizer by varying the stochastic variables involved in the optimizer.

Anirudh et al. [51] present the Wavefront Frontier Detector (WFD), an autonomous frontier-based exploration approach. It is described and put into practice on the Kobuki TurtleBot hardware platform and the Gazebo Simulation Environment utilizing the Robot Operating System (ROS). This algorithm’s benefit is that the robot can explore both huge, open areas and small, crowded areas. Additionally, the map produced using this method is contrasted and checked against the map produced using the turtlebot-teleop ROS package.

### Limitations

Multi-robot space exploration in situations with many obstacles is a difficult undertaking that calls for a thorough assessment of the techniques employed. We examine the drawbacks of both deterministic and swarm-based methods in the context of space exploration in this subsection.

**Deterministic Method:**

1. **Limited Robustness:** The behavior of the robots is frequently governed by specified rules and algorithms in deterministic approaches. This method’s capacity to deal with unforeseen circumstances or dynamic changes in the environment may be constrained in an environment that is dense with obstacles. The method might find it difficult to adjust to unforeseen impediments or disturbances, which can result in less-than-ideal or ineffective exploration.
2. **Lack of Scalability:** Deterministic approaches are usually developed for a fixed number of robots and a fixed number of obstacles. It can be difficult to scale up the system or accommodate varying obstacle densities. As the number of robots and obstacles rises, the deterministic nature of these algorithms may result in greater processing complexity and communication overhead.
3. **Exploration Efficiency:** Deterministic approaches frequently rely on specified exploration patterns or robot trajectories. While these patterns may be successful in some situations, they may not be optimized for obstacle avoidance or efficient environment covering. The inability to adjust and make real-time decisions may impede overall exploration efficiency.

**Swarm-based Method:**

1. **Lack of Determinism:** Swarm-based approaches frequently demonstrate emergent behavior that cannot be controlled explicitly. While adaptability might be beneficial, it can also pose difficulties in assuring deterministic and predictable behavior, particularly in complicated, obstacle-cluttered settings. Due to the lack of determinism, it can be difficult to ensure collision-free exploration or adherence to established mission objectives.
2. **Communication Overhead:** To achieve collective decision-making and work distribution, swarm-based approaches often necessitate substantial communication and coordination among the robots. In obstacle-cluttered environments where communication links can be broken or limited, relying on communication can impair overall system performance and scalability.
3. **Exploration Completeness:** Due to their reliance on local interactions and limited sensing capabilities, swarm-based approaches may fail to achieve thorough exploration of the environment. Certain environmental areas or regions may go unexplored or underexplored, resulting in insufficient mapping or data collection.

To summarize, both deterministic and swarm-based techniques for space exploration in obstacle-cluttered settings have flaws. Deterministic approaches may lack robustness, scalability, and exploration efficiency, whereas swarm-based methods may encounter determinism, communication-overhead, and exploration-completeness difficulties. To overcome these drawbacks, hybrid systems that integrate adaptability, scalability, efficient decision-making, and resilience to efficiently navigate and investigate obstacle-cluttered environments in space exploration missions are required.

## 3. Conceptualization of Parameterized Aquila Optimizer

We outline the framework for creating a parameterized Aquila Optimizer. It is first introduced and set up for a multi-robot configuration. After that, we elaborate step-by-step on how to formulate a parameterized Aquila Optimizer.

### Aquila Optimizer

Laith Abualigah et al. [52] introduced the Aquila Optimizer (AO), an algorithm with natural inspiration. The AO approach illustrates each step of the hunt’s activities to show an Aquila’s hunting habits. As a result, the suggested AO algorithm’s optimization operations are divided into four groups. A number of behavioral characteristics, as shown below, can shift the AO from exploration to exploitation:

**Stage 1: Expanded exploration**

As part of the first phase, the Aquila soars far above the earth before vertically diving once it has located its prey. The following is a mathematical depiction of this behavior:

(1) $$X_{1}\left(t+1\right)=X_{best}\left(t\right)\times \left(1-\frac{t}{T}\right)+(X_{M}\left(t\right)-X_{best}\left(t\right)\times rand\left(x\right)$$

(2) $$X_{M}\left(t\right)=\frac{1}{n}\sum_{j=1}^{n}X_{j}\left(t\right)$$

where $X_{best}\left(t\right)$ is the best location and

$X_{M}\left(t\right)$ means the positions of all Aquilas.
*T* is the value of the maximum iteration, *t* is the the current iteration,
*rand(x)* are random numbers from 0 to 1, and *n* is t the population of Aquilas.

**Stage 2: Narrowed exploration**

This is the method of hunting that the Aquila uses most frequently. The Aquila’s gliding assault is modeled as:

(3) $$X_{2}\left(t+1\right)=X_{best}\left(t\right)\times LF+X_{R}\left(t\right)-\left(y-x\right)\times rand(x)$$

where *rand(x)* is a random integer ranging from 0 to 1.

The Levy flight function is:

(4) $$LF=xx\times \frac{w\times vv}{|\upsilon {|}^{\frac{1}{\beta }}}$$

(5) $$vv=\left(\frac{⨿\left(1+\beta \right)\times sine\left(\frac{\pi \beta }{2}\right)}{⨿\left(\frac{1+\beta }{2}\right){\times \beta \times 2(}^{\frac{\beta -1}{2})}}\right)$$

where *w*, *v*, and *rand(x)* = [0,1] are random numbers, whereas *x* = 0.01, and β (constant) = 1.5. ⨿ is referred to as the gamma function. $X_{R}\left(t\right)$ is a randomly selected Aquila.

The parameters *yy* and *xx* are found in the following way to produce the spiral shape:

(6) $$\begin{array}{c} xx=rr\times \sin(\theta ) \end{array}$$

(7) $$\begin{array}{c} yy=rr\times \cos(\theta ) \end{array}$$

(8) $$\begin{array}{c} rr=rand(x)+U\times d \end{array}$$

(9) $$\begin{array}{c} \theta =\omega \times d+\frac{3\times \pi }{2} \end{array}$$

where *d* is the dimension of the search space, *U* is 0.005, ω is equal to 0.005, and rand(x) is has value between 1 and 20.

**Stage 3: Expanded exploitation**

The Aquila descends vertically to begin an initial attack once the general location of the target has been established using a third method. The AO uses the selected area to approach and attack the target. These actions are displayed as follows:

(10) $$\begin{array}{c} X_{3}\left(t+1\right)=\left(X_{best}\left(t\right)-X_{M}\left(t\right)\right)\times \alpha -rand\left(\right)+(\left(ub-lb\right)\times \\ rand(x)+lb)\times \gamma \end{array}$$

where *ub*, *lb* = 0 to 1, α and γ are small fixed numbers, and rand(x) is a random number.

**Stage 4: Narrowed exploitation**

The Aquila attacks its prey on the ground after pursuing its escape route. The mathematical representation of this behavior is:

(11) $$\begin{array}{c} X_{4}\left(t+1\right)=QF\times X_{best}\left(t\right)-\left(G_{1}\times X\left(t\right)\times rand\left(x\right)\right)\\ -G_{2}\times LF+rand(x)\times G_{1} \end{array}$$

where *QF* is a quality function and rand(x) is a random number.

(12) $$G_{1}=2\times rand(x)-1$$

(13) $$G_{2}=2\times \left(1-\frac{t}{T}\right)$$

(14) $$QF=t^{\frac{2\times rand(x)-1}{{\left(1-T\right)}^{2}}}$$

## 4. Integrated MAE-Parameterized Aquila Optimizer

The MAE-PAO formulation framework is provided in this part. Setting up the MAE for robotic configuration is the first step in the segment. The MAE-PAO structure is then finished by fusing the parameterized Aquila Optimizer with MAE.

### 4.1. Multi-Agent Exploration

During exploration, a group of autonomous agents works together to find what is missing. They surf in every corner in the region. They construct a map from the data they collect while exploring. Agents can choose from a wide variety of accessible communication algorithms.

This research focuses on a single application that factors in the price and the close-by distance that each robot must traverse. The ability to employ **Frontier cells** is the most valuable skill to have while venturing into uncharted territory. A frontier cell is a cell that has undergone exploration and is located next to a cell that has not yet been explored. The price of each cell fluctuates according to how far away it is from the robot’s starting place. The map’s evidence grids are employed for spanning. Each cell’s occupancy status is represented by a probability value. When the sensor model receives a new real-time sensor reading, it updates the grid accordingly. The robot creates a complete picture of its environment without any prior knowledge of it. The agent’s initial position on the map is fixed. The cost function establishes the gap between each agent and the subsequent frontier. In order to make an occupancy grid map, it is necessary to use the Robotics System Toolbox. Each cell’s price is determined by factors such as the sensor output, the Euclidean distance, and the occupancy likelihood.

(15) $$\begin{array}{cc} O_{x,y}=min\{O_{x+\Delta x,y+y}+\sqrt{\Delta x^{2}+\Delta y^{2}}. & \\ P(ocu_{x+\Delta x,y+\Delta y)}\} \end{array}$$

where Δx,Δyϵ$\left[-1,0,1\right]\;\Lambda \;P\left(ocu_{x+\Delta x},y+\Delta y\right)\epsilon \left[0,ocu_{max}\right]$ and $occ_{max}$ is the maximum probability value of the grid cell.

The cell is designated as a frontier cell, and its cost is saved for the preceding step if the sensor beam contacts it. Probabilities are used to check for obstacles in a cell. The probability of an unknown cell is 0.5, whereas it is 1 or 0 for an occupied or unoccupied cell, respectively. When the sensor beam approaches the cell from a given distance, the likelihood value lowers.

#### Utility Value

It is assumed that initially the utility of each cell on the map has the same value. See Equation (16) to see how these values shift as the agents go throughout the map. The agents are eager to investigate the high-utility cells on the map since they may hold information regarding previously unknown destinations.

(16) $$U_{j}^{c}=U_{j-1}^{c}-\Sigma P(\|ocu_{x,y}^{u}-ocu_{x,y}^{u^{\prime }}\left\|\right)$$

The utility value $U_{j}^{c}$ is equal to the last modification, represented as $U_{j-1}^{c}$, which can be altered by the selected robot or by previous robots before, and the likelihood of occupancy of the chosen cell is calculated by deducting the current robot’s location from the state of the previous changes. In iteration i, Equation (17) chooses the utility $U_{j}^{c}$ as the maximum value.

(17) $$\left(j,c\right)=max\left(U_{j}^{c}-V_{x,y}\right)$$

The agents complete the first iteration by beginning their search with path-scanning sensors. The target becomes less valuable because the process of spatial divergence accelerates. The ray length is preserved at 1.5 m, and the overall size is 20 × 20 m.

### 4.2. Parametrized Adaptable Aquila Optimizer

***G*_2_** is the stochastic variable found in the AO. It declines over the length of simulation runs. The original ***G*_2_** equation can be found in (13). We introduce an adjustment to ***G*_2_**. In addition to the *G*_2_ being updated adaptively, the ***gamma*** parameters are also added to regulate the loss of the *G*_2_ function. Modification is done as follows:

(18) $$\begin{array}{c} gamma=gamma_{min}+\left(gamma_{max}-gamma_{min}\right)\\ \times \frac{U_{max}-U_{min}}{f_{avg}-U_{min}};\;U_{max}\leq U_{avg}\\ gamma_{min}+\left(gamma_{max}-gamma_{min}\right)\\ \times \frac{U_{max}-U_{min}}{U_{max}-U_{avg}};\;U_{max}\geq U_{avg} \end{array}$$

If ***gamma*** is too small, the algorithm may get stuck in a local optimum, while if it is too large, the search may be insufficient and the optimal solution may not be found, limiting the effectiveness of the procedure. To achieve the optimal balance of **(*G*_2_)** between the algorithm’s search capabilities **(*QF*)** in the exploration and exploitation phases, it is helpful to integrate an adaptively updated **(alpha)**. In this post, we provide a formula similar to an equation that allows us to adjust the size based on the fitness value.

(19) $$gamma^{-}=1-gamma\left(t\right)+eps$$

(20) $$\begin{array}{c} G_{2new}=2\times {\left(1-\frac{t}{T}\right)}^{gamma} \end{array}$$

where (*t*) stands for the current value; min and max stand for the minimum and maximum values, respectively, in terms of the experience value; *U_min_*, *U_max_*, and *U_avg_* stand for the minimum, maximum, and average fitness values, respectively, of utility values of the cell at the current iteration; and eps stands for the effective parameter size. Replacement of the old value with the adaptively updated one speeds up the search and improves convergence. Refer to the new Equation (13).

### 4.3. Integrated MAE-Parametrized Adaptable Aquila Optimizer

Agent mobility with sensor coverage is used to study an unknown area in the MAE-PAO’s formulation. In the literature, static sensors are typically employed to cover a large region in an unknown environment [53]. The suggested method starts by utilizing robots to create (an) environment map(s). The map construction is done using grid occupancy via utilizing the Robotic Operation Systems Toolbox (ROS) in MATLAB. Deterministic exploration integrated with the Aquila method helps the agent settle on a new location. The proposed algorithm clarifies the MAE-parametrized adaptable Aquila investigation. Refer to Algorithm 1.
**Algorithm 1** Hybridized MAE-Parametrized Aquila Optimizer1:Set robot number, iteration, and agent start position2:utility = 13:**while** iter =iter+1 **do**4:   **for** all robot numbers **do**5:     Initialize coordinates of $O_{cell}$6:     Determine cost of $O_{cell}$7:     Subtract $U_{c}^{j}$ & $O_{cell}$8:     Use *X*_1_, *X*_2_, *X*_3_, and *X*_4_,9:     Upgrade X(iter+1) utilizing Equations (1), (3), (10) and (11)10:     Choose agent position again11:     Minimize utility12:   **end for**13:   Determine *G*_1_, *G*_2_′, and *QF*′14:**end while**15:Return the solution

The utility can have a maximum value of 1, which is possible. The agent’s sensor cell is divided into eight 8-vector cells, or $C_{s}$, with each cell consisting of $C1_{x,y},C2_{x,y}\ldots \ldots ,C8_{x,y}$. The cells are thought to be plausible contenders for the jobs. The X(iter+1) location is updated using Equations (1) and (11) in the suggested method. Using Equation (17), the technique then calculates the cost for the eight cells and subtracts the various utility values from the cost. The priorities of the vetted cell alter as a result of $G_{1},G_{2}$, *QF*, and the occupancy probability values of the dominated cells. The dynamic parametric characterization $t\leq (\frac{2}{3}\times T)$ is utilized to distinguish between the exploration and exploitation phases. With the aid of the random number r1, the phases are changed. The suggested approach tries to converge the *G*_1_, *G*_2_, and Quality Functions.

(21) $$\begin{array}{c} X_{1}\left(t+1\right)=P_{au,k}\left(ocu_{x+\Delta x,y+\Delta y}\right)\\ \times \left(1-\frac{t}{T}\right)+\left(X_{M}\left(t\right)-P_{k}\left(ocu_{x+\Delta x,y+\Delta y}\right)\times r1\right) \end{array}$$

(22) $$\begin{array}{c} X_{2}\left(t+1\right)=P_{au,k}\left(ocu_{x+\Delta x,y+\Delta y}\right)\times LF\left(D\right)+\\ P_{au,k}^{R}\left(ocu_{x+\Delta x,y+\Delta y}\right)-\left(y-x\right)\times rand\left(\right) \end{array}$$

(23) $$\begin{array}{c} X_{3}\left(t+1\right)=P_{au,k}\left(ocu_{x+\Delta x,y+\Delta y}\right)-X_{M}\left(t\right))\times \alpha \\ -rand()+((ub-lb)\times rand()+lb)\times \delta \end{array}$$

(24) $$\begin{array}{c} X_{4}\left(t+1\right)=QF^{\prime }\times P_{au,k}\left(ocu_{x+\Delta x,y+\Delta y}\right)-(G_{1}\times \\ P_{k}\left(ocu_{x+\Delta x,y+\Delta y}\right)\times rand\left(\right))\\ -G_{2}^{{}^{\prime }}\times LF\left(D\right)+rand\left(\right)\times G_{1} \end{array}$$

The most important thing to keep in mind is that we have not used the mean value from Equations (1) and (10) because it is related to the intelligence-dominance-agent-using behavior of the Aquila. Finding the average robot placements among the different Aquila agents is not necessary for the target selection problem.

The robot’s next-best position *X*(*t* + 1) is transmitted automatically to the best Aquila operator, who is subsequently rewarded with the highest value. The utility values of neighboring cells are thus lowered using Equation (16). By generating fresh random values for the next iteration, $G_{1},G_{2}$, and QF push the process closer and closer to a converged state. The upcoming ideal Aquila Operator is enhanced in value with each iteration of the hybrid MAE-PAO. The pros and cons of a robot’s surrounding grid cells are taken into account. *G*_1_, *G*_2_, and *QF* vote on the best operator. This means that the agent has to use the cell value anticipated by the occupancy probability to choose its next move. The robot uses MAE-PAO for the upcoming maneuver. Uncharted regions of the map provide more information than studied cells or regions. The robot is attracted more to high-utility cells when the utility value is split by the cost of the known cells. Maximum values become attractive targets for future robot locations if the utility cost of the unfamiliar cells is subtracted from the costs with the lowest values. In contrast, the proposed hybrid stochastic method provides four alternatives for optimizing the reordering of hierarchies across a range of stochastic parameters. Value may be best protected through prolonged investigation or broadened exploitation, as opposed to the more restricted approaches taken by the CME.

## 5. Discussion of Simulation Results

In this section, we show the results of the multi-coordinated investigation that is proposed utilizing the Aquila method. The feasibility of the proposed method is evaluated by increasing the complexity of the map. The number of challenges is adjusted for variety. The map always has a 20 m × 20 m size. The robotics toolbox is used to create the maps. The space is separated into an open area that needs to be explored and a dark zone that denotes the region where barriers are present. The outcomes are contrasted with multi-agent-Aquila and multi-agent-whale optimizers in order to validate them and determine whether there are any advantages.

To calculate the total area of the investigated cells, we use Equation (25).

(25) $$Total\;area=\frac{Unsurfed\;area\;-Surfed\;area}{Surfed\;area}$$

This characteristic is used by the multi-agent to assess the area it will be surfing. A value of 0 indicates that there is no area being researched, while a value of 1 indicates that the entire region has been examined. The resulting figure will serve as an evaluation criterion for the percentage of the area under investigation.

**Figure 1:**
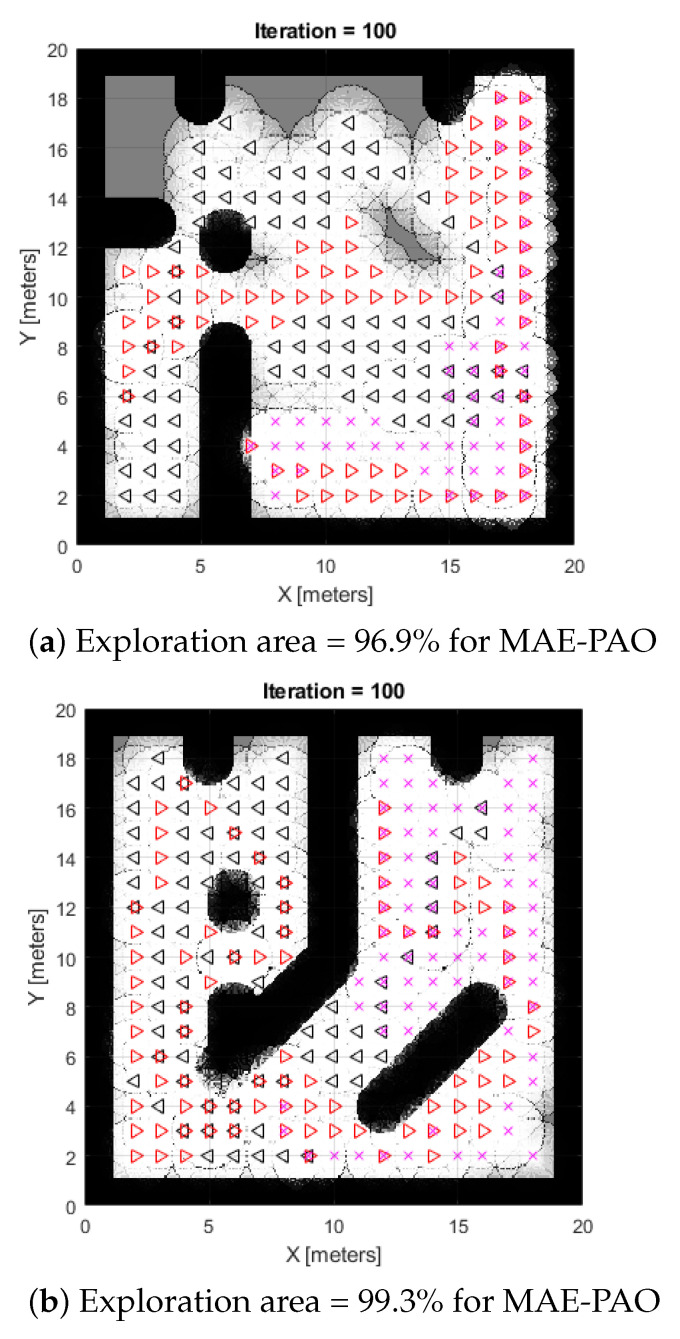
Simulation of CME-parametrized Aquila exploration algorithm.

The framework is then tested using two alternative scenarios, as shown in Figure 1a,b. The vastness of the map, coupled with the total number of obstacles, iterations, and robot locations, is one of its most visible features. The results show that in just 27 and 29 s, respectively, 96.9 and 99.3 percent of the total area was successfully probed. Additionally, more iterations can lead to even better performance characteristics. The results provide strong evidence for the effectiveness of the suggested strategy because it not only allowed for extensive searches of a wide area but also significantly decreased computer complexity and exploration time frames. Hence, MAE-PAO successfully illustrates quick and effective map exploration.

## 6. MAE-PAO Algorithm Compared to the Latest CME-AO and Whale Algorithms

Our proposed MAE-PAO’s performance has been thoroughly simulated across a wide range of environmental conditions, and now we are taking those results and running with them. Here, we look at the outcomes from both MAE-PAO and the state-of-the-art CME-AO (CME-Aquila Optimizer) and whale technique, comparing and contrasting them using in-depth research and analysis. The study takes place in a more complex setting that presents challenging situations and a plethora of obstacles that appear in random order. We look at how the system operates in the two different environmental conditions that Maps 1 and 2 reflect. These maps’ complexity varies depending on the direction, number, and length of the barriers. The comparison accurately accounts for all significant variables, such as the percentage of the map that was explored, the total number of runs that were abandoned, and the cumulative time needed to explore the maps under varied conditions.

**Figure 2:**
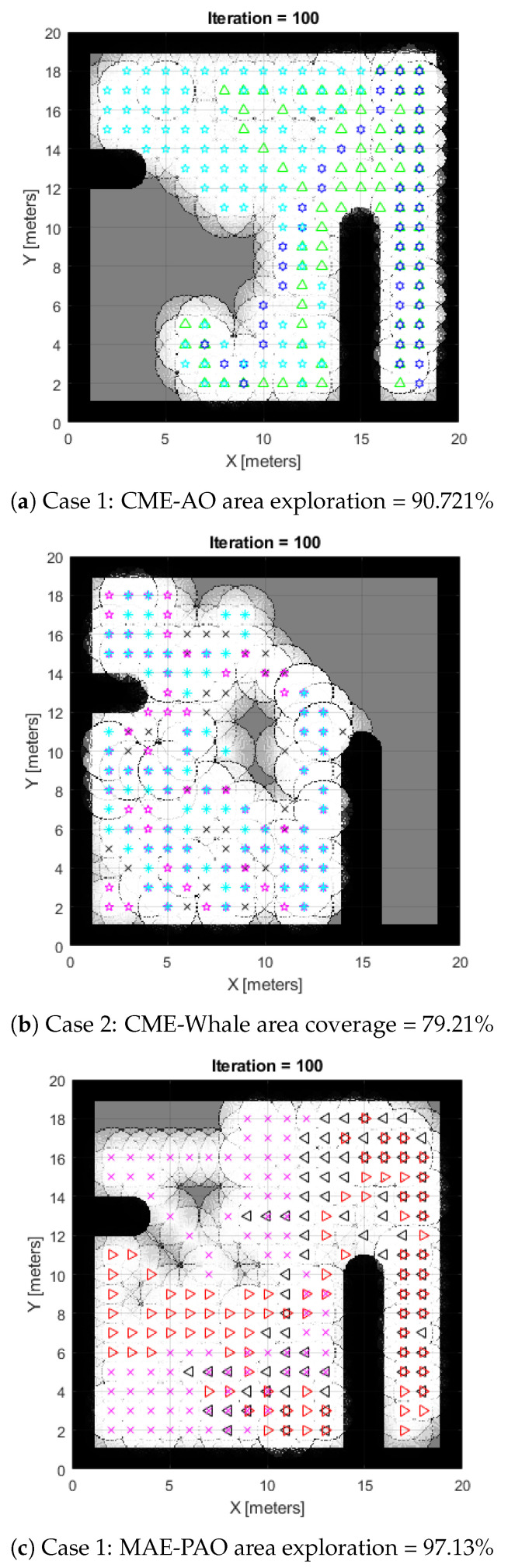
Case 1: Results of CME-AO, whale, and MAE-PAO exploration algorithms.

**Case 1:** The use of a classic Aquila Optimizer with multi-agent exploration (CME-AO) is shown in Figure 2a, while our suggested Adaptive Aquila Optimizer (MAE-PAO) is also visible in Figure 2b. According to the simulation results, the traditional Aquila Optimizer covers 90.721% of the region in about 40.2 s with one failed run. The proposed MAE-PAO examined 97.13% of the area in around 25.12 s with one failed run, which is a substantially shorter amount of time. The deterministic approach’s inherent nature, which necessitates the agent takes the same path each time the simulation starts, means that CME cannot fully explore the map on its own. Increasing the total number of iterations also improves computing capability.

**Figure 3:**
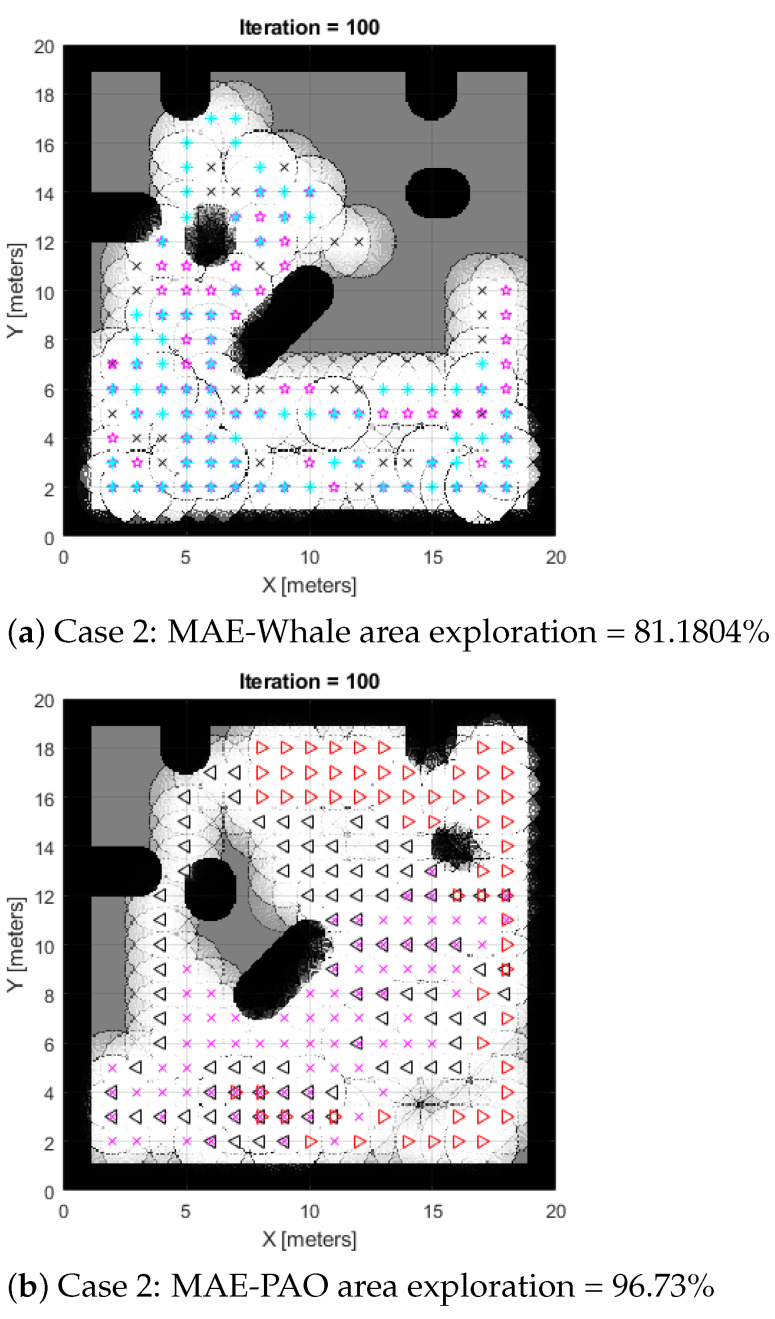
Case 2: Results of CME-AO, whale, and MAE-PAO exploration algorithms.

**Case 2:** The comparative findings between the proposed MAE-PAO and the traditional Aquila under the various environmental circumstances shown in Map 2 are shown in Figure 3a,c. A total of 96.73% of the environment was explored by the suggested MAE-PAO algorithm. The required time was 27.13 s, and there were zero unsuccessful runs. With the traditional Aquila Optimizer, a similar exploration rate of 94.41 percent was attained. The algorithm needed to be run numerous times, and each run took 45.7 s to complete. This shows that although the exploration rates of the two algorithms are comparable, the suggested MAE-PAO’s rate of execution and the number of failed simulated runs are much lower, providing more persuasive evidence of the algorithm’s effectiveness.

### 6.1. Summarized Results

For quick reference and the readers’ convenience, the whole set of results described above is condensed in Table 1. The results obtained using the suggested MAE-PAO and the referred CME-AO algorithms are shown in the cited Table 1. The comparison properly takes into account all relevant factors, including the proportion of the entire area that was investigated, the number of failures, and the amount of time needed for map exploration in various situations. According to the findings, the primary goal of space exploration is satisfactorily accomplished by the suggested MAE-PAO approach in fewer test runs and less time. The traditional CME-AO algorithms have a modest propensity to investigate simpler environments. Additional runs and exploration times are negative. As a result, the suggested MAE-PAO algorithm is a popular option for onboard practical use.

### 6.2. Statistics-Based Performance Evaluation

Here, we compare the proposed MAE-PAO to CME-Aquila and whale in terms of performance by analyzing the two systems’ respective statistical properties. Multiple tests in the same environmental conditions are done in order to conduct the inquiry, as demonstrated in Figure 2 and Figure 3, respectively. We examine the mode and dispersion of the percentages, and we compute the full duration of the process. The findings acquired across several runs for the two separate environmental conditions indicated by Maps 1 and 2, respectively, are shown in Table 2 and Table 3.

For the proposed algorithm MAE-PAO (refer to Table 2), the average mean exploration rate for Case 1 and Case 2 is 97.15%. The average mean time consumed by MAE-PAO is 26.41 s. Similarly, for algorithms CME-Aquila and whale, refer to Table 3 and Table 4, respectively, which depict similar salient details.

The collective rates of exploration along with the time taken for the exploration of the proposed MAE-PAO and the contemporary CME-Aquila and whale algorithms are jotted in Table 5. As mentioned in the provided table, the proposed MAE-PAO has an average area exploration rate of approx 97.15%, which is greater than the area exploration rates of 92.76% and 80.452% for CME-Aquila and whale, respectively. Similarly, the mean (26.41 s) exploration time of the proposed MAE-PAO is much less than those of CME-Aquila (43.32 s) and whale (31.164 s). This empirically verifies the superior efficiency of MAE-PAO’s exploration across a broad variety of environmental situations. There is also very little variation across runs in terms of the exploration rate and exploration time.

The whale algorithm is presented with the CME method to further validate the proposed algorithm. The comparison of the whale algorithm demonstrates that it underperforms as compared to the proposed algorithm. The method was statistically tested for an average rate of exploration and for the time taken to complete the exploration process. The number of iterations was kept at 100 because the environment dimension is 20 × 20, which requires no more than 100 runs. If we increase the number of runs, the robots/agents keep on exploring the area that has already been explored, and decreasing the number of runs results in inefficient and insufficient area exploration. The statistic results are mentioned in Table 4. The average rate of exploration and time was found to be 80.3440% and 31.1760 s, respectively.

### 6.3. CME-AO Test on Additional Map

The same idea is extended to a map containing different-shaped obstacles that are added to check the efficacy of the algorithms. Since the robots are exploring the map on the ground level, the height factor is ignored. Secondly, the size of the obstacle does not affect the rate of exploration in a map. The number of robots is kept at three; the algorithm is designed so that all three robots collaborate simultaneously to share information about their whereabouts on the map during the process of exploration. The Robotic System Toolbox is employed to create a 2D grid map. This is why the shape of the map is constant in the algorithm, whereas colors refer to different robots.

**Figure 4:**
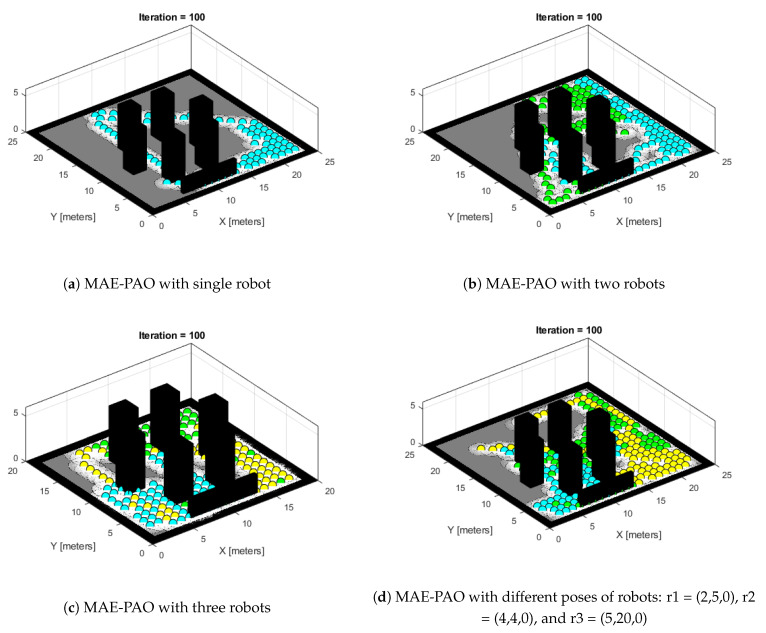
MAE-PAO exploration process over different map configurations: r1 = (6,5,0), r2 = (7,4,0), and r3 = (8,4,0).

The robot poses are specified as follows: r1 = (6,5,0), r2 = (7,4,0), and r3 = (8,4,0) for the first three in Figure 4a–c, and r1 = (2,5,0), r2 = (4,4,0), and r3 = (5,20,0) for Figure 4d. The map dimensions are 20 × 20 m for the first two figures and 25 × 25 m for Figure 4c. These configurations are employed to examine the effectiveness of the robot’s exploration process in various map configurations.

Figure 4a demonstrates the exploration process with a single robot, Figure 4b illustrates two robots, and Figure 4c showcases the exploration process with three robots. Lastly, Figure 4a depicts the exploration process with the same number of robots but with different robot poses.

The purpose of presenting these experimental data is to evaluate the effectiveness of the proposed algorithm under various map configurations and robot positions. This adds to the study’s contribution by providing insights about the algorithm’s adaptability and performance in a variety of scenarios.

## 7. Conclusions

The study presented a novel approach to exploring unknown environments with multiple robots. The internal variable of the Aquila Optimizer was modified to function to improve the search capabilities in the suggested algorithm. The implementation of the Parameterized Aquila Optimizer enabled the robots to effectively coordinate and adapt to changing environmental conditions while maximizing exploration efficiency. Fast convergence was achieved by the hybrid approach’s fine-tuning of the optimizer’s parameters. The inherent benefits, as demonstrated in Section 6, include increased map exploration in a crowded environment, drastically shortened execution time, and the fewest possible failed runs. The performance of the algorithm was evaluated utilizing parametric checks and statistical parameters. For the proposed MAE-PAO, the mean rate of exploration (97.15%) was much greater than those of the other algorithms (92.76% and 80.452% for CME-Aquila and CME-Whale, respectively). The mean execution times were 26.41 s, 43.32 s, and 31.164 s for MAE-PAO, CME-Aquila, and CME-Whale, respectively. Future work directions in this area could involve extending the proposed method to handle more complex environments, integrating additional sensory information, and incorporating other optimization algorithms to further improve the exploration performance. Additionally, incorporating decentralized decision-making strategies and addressing scalability issues in larger multi-agent systems could also be interesting areas of future research.

## Figures and Tables

**Table 1:** Performance comparison: CME-Aquila, Whale, and MAE-PAO.

Map No	CME-Aquila			CME-Whale			MAE-PAO		
	% Area Explored	Failures	Run-Time (s)	% Area Explored	Failures	Run-Time (s)	% Area Explored	Failures	Run-Time (s)
Case 1	90.721%	1	40.2	79.21%	2	31.164	97.13%	1	25.12
Case 2	94.41%	2	45.7	81.1804%	4	33.21	96.73%	0	27.13

**Table 2:** Statistical values of exploration rates and execution times for Maps 1 and 2 for MAE-PAO; refer to Figure 2c and Figure 3c.

Map	Exploration Rate	Exploration Time	Average Mean	Average Time
Map1	Run1 = 97.56%	Run1 = 25.9	97.1510%	26.41502
	Run2 = 96.87%	Run2 = 24.9		
	Run3 = 97.87%	Run3 = 25.3		
	Run4 = 97.54%	Run4 = 25.69		
	Run5 = 96.77%	Run5 = 25.7		
Map 2	Run1 = 97.58%	Run1 = 27.17		
	Run2 = 97.12%	Run2 = 26.9		
	Run3 = 96.32%	Run3 = 27.19		
	Run4 = 96.87%	Run4 = 27.98		
	Run5 = 97.01%	Run5 = 27.5		

**Table 3:** Statistical values of exploration rates and execution times for Maps 1 and 2 for CME-Aquila; refer to Figure 2a and Figure 3a.

Maps	Exploration Rates	Exploration Time	Average Mean	Average Time
Map 1	Run1= 90.25%	Run1= 40.87	92.762%	43.3250%
	Run2 = 91.13%	Run2 = 41.13		
	Run3 = 90.86%	Run3 = 41.98		
	Run4 = 91.11%	Run4 = 41.55		
	Run5 = 90.5%	Run5 = 40.83		
Map 2	Run1 = 94.78%	Run1 = 45.32		
	Run2 = 93.96%	Run2 = 46.12		
	Run3 = 94.74%	Run3 = 45.3		
	Run4 = 94.3%	Run4 = 44.9		
	Run5 = 95.74%	Run5 = 45.4		

**Table 4:** Statistical values of exploration rates and execution times for Maps 1 and 2 for CME-Whale; refer to Figure 2b and Figure 3b.

Maps	Exploration Rates	Exploration Time	Average Mean	Average Time
Case 1	Run1 = 79.21%	Run1 = 33.65	80.452%	31.164%
	Run2 = 80.62%	Run2 = 32.85		
	Run3 = 79.89%	Run3 = 30.87		
	Run4 = 80.25%	Run4 = 30.03		
	Run5 = 78.65%	Run5 = 28.48		
Case 2	Run1 = 81.18%	Run1 = 33.21		
	Run2 = 78.32%	Run2 = 27.38		
	Run3 = 80.24%	Run3 = 32.64		
	Run4 = 80.98%	Run4 = 30.87		
	Run5 = 81.99%	Run5 = 31.66		

**Table 5:** Collective means and times for all MAE-PAO, CME-Aquila, and CME-Whale (as depicted in Table 2 and Table 3).

Algorithm	Total Rate Mean	Total Mean Time
MAE-PAO	97.15%	26.41%
CME-Aquila	92.76 %	43.32%
CME-Whale	80.452%	31.164%

## Data Availability

All data used in this research are available upon request.
